# Increased Diversity and Introduction of Multidrug-Resistant Strains of *Neisseria gonorrhoeae* Following Cessation of COVID-19 Pandemic–Related Travel Restrictions: An Observational Genomic Epidemiologic Study

**DOI:** 10.1093/infdis/jiag097

**Published:** 2026-02-12

**Authors:** Rosa C Coldbeck-Shackley, Erin Flynn, Arshdeep Kaur Mudhar, Mona L Taouk, George Taiaroa, Charlotte Bell, Trisha J Rogers, Caitlin A Selway, Lito Papanicolas, Mark Turra, Lex E X Leong

**Affiliations:** Microbiology and Infectious Diseases, SA Pathology, Adelaide; Microbiology and Infectious Diseases, SA Pathology, Adelaide; Microbiology and Infectious Diseases, SA Pathology, Adelaide; Department of Infectious Diseases, The University of Melbourne at the Peter Doherty Institute for Infection and Immunity; Department of Infectious Diseases, The University of Melbourne at the Peter Doherty Institute for Infection and Immunity; Department for Health and Wellbeing, Government of South Australia; Department for Health and Wellbeing, Government of South Australia; Microbiology and Infectious Diseases, SA Pathology, Adelaide; Microbiology and Infectious Diseases, SA Pathology, Adelaide; Microbiology and Infectious Diseases, SA Pathology, Adelaide; Microbiology and Infectious Diseases, SA Pathology, Adelaide; UniSA Clinical and Health Sciences, University of South Australia, Adelaide

**Keywords:** antimicrobial resistance, *Neisseria gonorrhoeae*, public health, surveillance, whole genome sequencing

## Abstract

**Background:**

National and international travel drives the spread of antimicrobial resistance in high-priority pathogens, including *Neisseria gonorrhoeae*. Border closures and travel restrictions in response to the COVID-19 pandemic had wide-reaching impacts on infectious disease epidemiology, including the transmission and genomic diversity of *N gonorrhoeae.* However, less is known about *N gonorrhoeae* population structures in the years following the lifting of pandemic restrictions.

**Methods:**

This study analyzed *N gonorrhoeae* genomic data collected for routine public health surveillance in South Australia, Australia, and contextual sequences from Victoria, Australia, before and after the cessation of COVID-19 interstate and international travel restrictions.

**Results:**

*N gonorrhoeae* was highly clonal during periods with restricted travel, and genomic diversity markedly increased after restrictions were removed, possibly driven by increased transmission and the introduction of new strains.

**Conclusions:**

Routine genomic surveillance is an important public health tool for the monitoring of *N gonorrhoeae*, especially the introduction and spread of antimicrobial resistant strains.

## Teaser

*Neisseria gonorrhoeae* genomic diversity rebounded after lifting of the COVID-19 travel bans in South Australia due to increased local diversification and new strain introductions. Identification of 2 new multidrug resistance strains after border opening highlights the need for ongoing genomic surveillance.

Gonorrhea is a sexually transmitted infection (STI) caused by the bacterium *Neisseria gonorrhoeae* (NG). If left untreated, gonorrhea can cause serious complications, including pelvic inflammatory disease and adverse pregnancy outcomes in women, epididymo-orchitis in men, and infertility and increased risk of HIV infection in women and men [1]. According to the World Health Organization, the recommended treatment for uncomplicated NG infection is ceftriaxone (CTX) monotherapy or CTX/azithromycin (AZI) dual therapy. However, the increased prevalence and spread of antimicrobial resistance (AMR) and multidrug resistance (MDR) are compromising the effectiveness of these first-line treatments, raising the threat of untreatable NG infections [2, 3].

Border closures and travel restrictions in response to the COVID-19 pandemic had wide-reaching impacts on global infectious disease epidemiology, including gonorrhea. In Europe and Australia, the introduction of these measures was associated with decreased NG genomic diversity [4–6]. However, less is known about changes to NG population structures and AMR following the cessation of COVID-19 restrictions and the subsequent resurgence of NG cases [7, 8].

In the Australian jurisdiction of South Australia (SA), COVID-19 restrictions introduced in March 2020 included intermittent closure of state borders until 23 November 2021 and sustained closure of the international border until 21 February 2022 [9, 10]. This study presents genomic surveillance data from NG isolated from notified gonorrhea cases in SA between October 2021 and March 2024, prior to and following cessation of COVID-19 travel restrictions. Here, we aimed to assess NG population changes after the resumption of interstate and international travel and to investigate the utility of routine genomic surveillance to detect and monitor MDR gonorrhea.

## METHODS

### Samples and Epidemiologic Metadata

SA Pathology is the NG referral laboratory for SA, receiving all cultured NG from diagnostic laboratories within the state. Across Australia, the nucleic acid amplification test is the most used diagnostic test for gonorrhea, and culture is performed only when requested by the referring clinician. Since September 2021, all cultured NG isolates from notified gonorrhea cases have been sequenced as part of routine surveillance. Genome sequences pertaining to samples collected between 1 October 2021 and 31 March 2024 were included in the study (n = 1208). Sequences from cases below the age of consent in SA (<17 years) and duplicate samples were excluded from the dataset. Duplicates were defined as isolates from the same patient with matching multilocus sequence type (MLST) and collection dates within 1 month.

Epidemiologic metadata associated with cases were collected from the state Notifiable Infectious Diseases Surveillance system. Sex was recorded as sex assigned at birth, and the lookback period for risk factors was 12 months.

### Isolate Culture, Whole Genome Sequencing, and Phenotypic Resistance Profiling

NG samples collected on liquid Amies swabs received by SA Pathology's bacteriology laboratory were plated onto split selective solid media Micro Neisseria Medium/Chocolate Agar (NIMM/CHOC Agar; Edwards Group Pty Ltd) and incubated at 38 °C with 5% CO_2_. At 48 hours, plates were examined for the presence of *Neisseria*-like colonies, which were confirmed with MALDI-TOF mass spectrometry (Bruker Daltonics Inc). Following confirmation of NG growth, a single colony was subcultured onto CHOC agar (38 °C with 5% CO_2_) for 48 hours to obtain a pure isolate. Subsequent DNA extraction and sequencing library preparation for whole genome sequencing were performed as previously described [11].

For susceptibility testing, isolates were cultured on GC Sensitivity Test Agar (Thermo Fisher). Penicillin (PEN) and ciprofloxacin (CIP) sensitivity was determined via the disc diffusion method of the Clinical and Laboratory Standards Institute [12]. Minimal inhibitory concentrations (MICs) for AZI and CTX were obtained with ETEST (bioMerieux). Resistance interpretations were defined by clinical breakpoints per the Clinical and Laboratory Standards Institute [13]. The Australian Alert System for Critical Antimicrobial Resistances definition (MIC >1 µg/mL) was used for AZI low-level resistance [14]. Additionally, CTX “reduced susceptibility” was defined as MIC ≥0.064 µg/mL as previously described [15, 16]. High-level or intermediate tetracycline (TET) genotypic resistance was identified by detection of the plasmid-mediated *tetM* gene [17] or the chromosomal-mediated *rpsJ* V57 M substitution [18] with ABRicate or ARIBA, respectively [19, 20].

### Genomic Analyses and Quality Control Assessment

Base calling was performed in bcl2fastq (version 2.19.0.316), followed by quality control assessment, de novo assembly, and molecular characterization via the in-house pipeline PHEsiQCal (version 3.0.0). Briefly, read quality was assessed in seqtk (version 1.3-r106 [21]). Taxonomic classification was performed with Kraken2 (version 2.1.2; database: k2_pluspf_20220607 [22]). Sequenced reads were de novo assembled per the Shovill pipeline (version 1.1.0 [23]) with the maximum depth set to 80. Assemblies were filtered through seqtk for sequences <500 base pairs, followed by quality assessment with the *fa* function from the Nullarbor pipeline (version 2.0.20191013 [24]).

MLST analysis was performed on filtered assemblies in mlst (version 2.23.0; database: pubMLST v2023-09-28 [25]). AMR was assessed by multiple tools on default settings: ARIBA (version 2.14.6; database: CARD version 3.2.7 [20]) and ABRicate (version 1.0.1; database: CARD version 2023-10-06 [19]). NG sequences passed quality control assessment if they had a minimum of 800 000 reads, <500 contigs, and top species identification of “*Neisseria gonorrhoeae*.” NG sequences that passed quality control were further analyzed for AMR type according to *N gonorrhoeae* Sequence Typing for Antimicrobial Resistance (NG-STAR) based on 7 genes (*penA*, *mtrR*, *porB*, *ponA*, *gyrA*, *parC*, and 23S rRNA) in pyngSTar (version 2021-10-25; database: version 20230915 [26, 27]) as part of the in-house typing analysis pipeline PHET (version 3.0.0).

### Core Genome MLST Phylogenetic and Cluster Analysis

The core genome MLST (cgMLST) scheme utilized PubMLST *N gonorrhoeae* cgMLST version 1.0 that was adapted for chewBBACA (version 3.3.6) [28–30]. This scheme was refined by using all SA sequences that passed quality assessment; chewBBACA AlleleCall outputs were extracted with a 95% presence threshold, including approximately 1500 core loci (see Supplementary Figure 1 for scheme validation data). Sequence relatedness was analyzed by extracted allele matrices as input for cgmlst-dists (version 0.4.0) analysis, and single-linkage hierarchical clustering was performed with the R-hclust function (version 4.2.2) [31, 32]. Cluster identity was assigned for clusters with ≤30 allelic differences (*h*30) using *cg*C- nomenclature. Relatedness was visualized in GrapeTree (version 2.2) with the MSTreeV2 model.

For context, cgMLST analysis was conducted with all SA sequences that passed quality assessment and 3714 publicly available genomic sequences [28] from a neighboring jurisdiction, Victoria (VIC), with collection dates of July 2019 to June 2021 (see Supplementary Figure 2 for phylogeny).

### Statistical Analysis and Data Visualization

A Wilcoxon rank sum (*W*) test was used for continuous data comparison between 2 groups with nonparametric distributions. χ^2^ tests were performed for comparisons between categorical data. All statistical analyses were performed in R (version 4.2.2).

## RESULTS

### Sample Demographics and Representativeness

During the study period, 1208 NG isolates were sequenced, and 1117 were unique samples from individual notifications. Of these, 1108 passed whole genome sequencing quality assessment and were included in subsequent analyses, representing 22.38% of gonorrhea notifications in SA.

**Figure 1.**
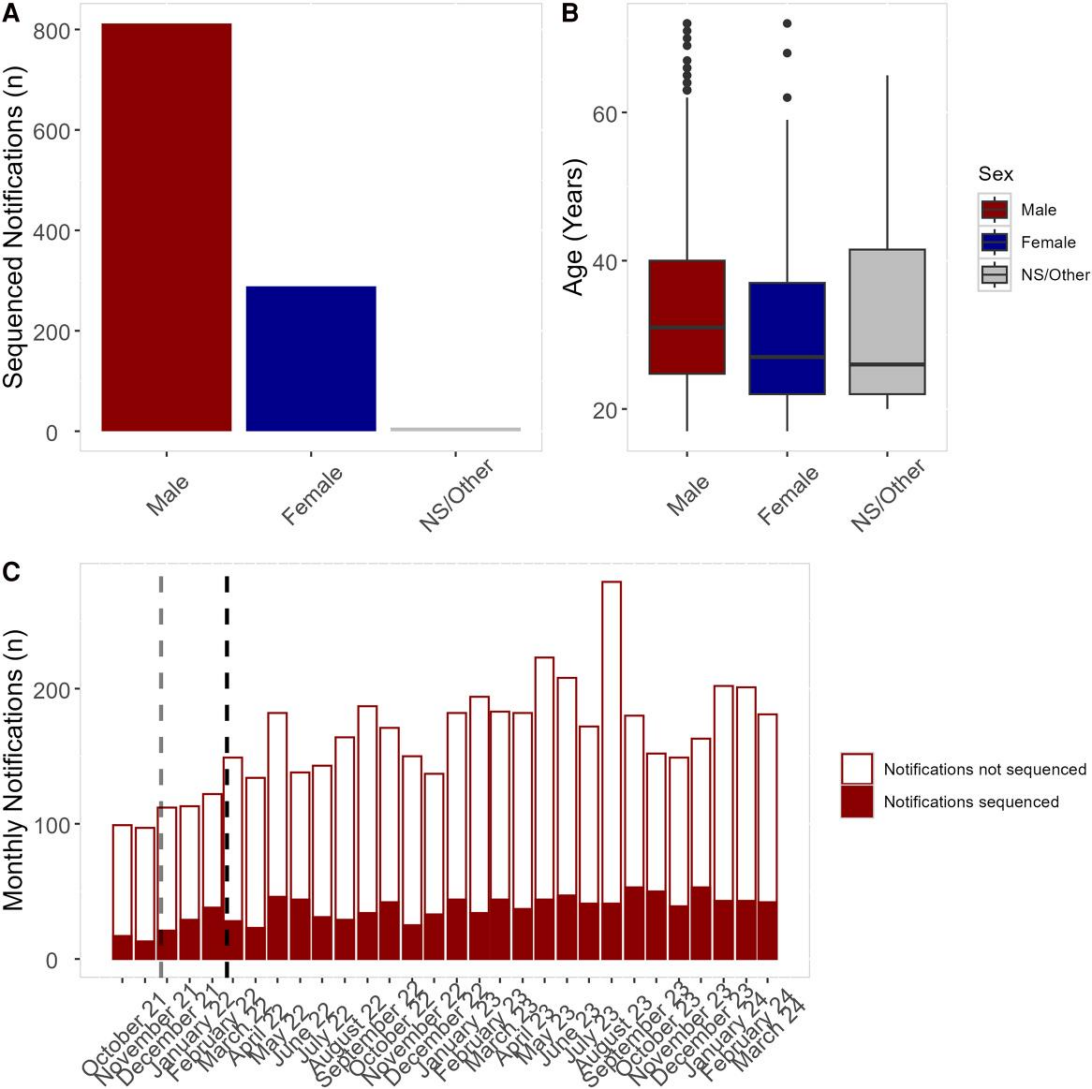
Demographics of *Neisseria gonorrhoeae* notifications sequenced during the study period representing unique infection incidence that passed whole genome sequencing quality assessment. *A*, Sequenced isolates from male, female, and no stated or other (NS/other) sex notifications. *B*, Ages of male, female, and NS/other sex notifications. Data are presented as median (line), IQR (box), and the smallest or largest observations 1.5 x IQR (error bars). *C*, *N gonorrhoeae* isolates sequenced as compared with the number of gonorrhea notifications in South Australia during the study period. Opening of state borders (23 November 2021) is indicated by the gray dashed line; opening of international borders (21 February 2022) is indicated by the black dashed line.

Females represented 26.08% of the sequenced isolates (n = 289), significantly less than males (n = 812, 73.22%; χ^2^ = 1276.5, *P* = 2.2e-16; Figure 1*A*). Seven people had no stated or other sex (0.6377%). Median ages were 31, 27, and 26 years for males, females, and no stated/other, respectively, and males were significantly older than females (*W* = 3164, *P* = 1.73e-06; Figure 1*B*). Isolates were derived from clinical samples collected at a range of health services (Supplementary Figure 3), and the proportion of notifications sequenced was stable during the study period (minimum, 13.40%; maximum, 32.89%; mean ± SD, 22.53% ± 5.28; Figure 1*C*). The mean number of monthly notifications prior to the state border opening (October and November 2021) was 98 as compared with 165 for the remaining study period (68.36% increase).

### Overview of NG Population in SA During the Study Period

**Figure 2.**
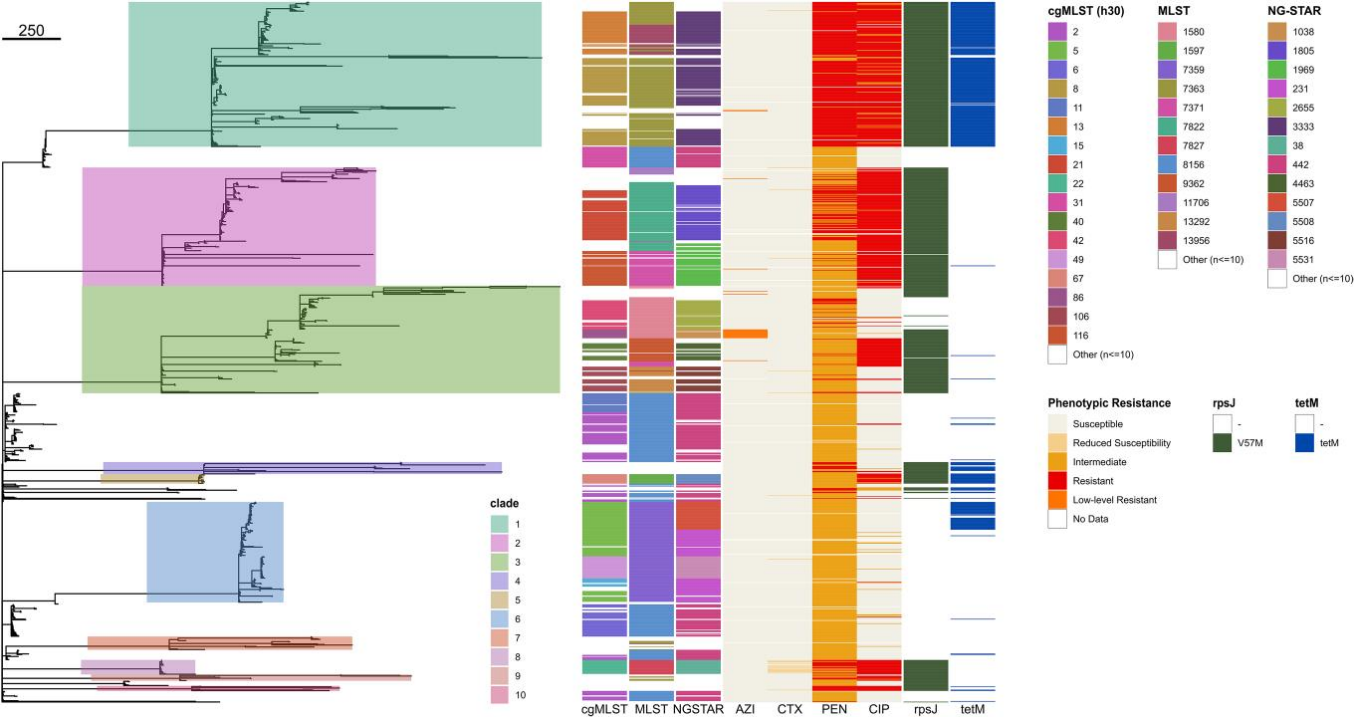
The *Neisseria gonorrhoeae* population in South Australia. Phylogenetic tree built from the cgMLST 95% threshold allele matrix via the GrapeTree MSTreeV2 model. Only the most common cgMLST (*h*30), MLST, and NG-STAR types (n > 10) are colored for simplicity. Phenotypic resistance interpretations for azithromycin (AZI), ceftriaxone (CTX), penicillin (PEN), and ciprofloxacin (CIP) were derived from the minimal inhibitory concentrations. Genomic markers of tetracycline intermediate- and high-level resistance (*rpsJ* V57 M and *tet*M, respectively) are also indicated. The tree scale represents the number of locus differences. Abbreviations: cgMLST, core genome multilocus sequence type; MLST, multilocus sequence type; NG-STAR, *N gonorrhoeae* Sequence Typing for Antimicrobial Resistance.

The NG population spanned multiple phylogenetic clades, mostly aligning with MLST sequence type (ST) groupings (Figure 2). However, ST-8156 isolates were split across clades and cgMLST (*h*30) groups, confirming that MLST alone cannot determine NG clonality due to horizontal gene transfer events of the MLST loci [29]. A total of 59 MLSTs and 113 NG-STAR unique STs were identified during the study period. The most common MLSTs observed were ST-8156, ST-7363, and ST-7359 (n = 245, 182, and 160), and the most common AMR NG-STAR STs (*ng*STs) were *ng*ST-442, *ng*ST-3333, and *ng*ST-1805 (n = 215, 156, and 76).

PEN and CIP resistance was widespread and most prevalent in clade 1 and clades 1 and 2, respectively (Figure 2; PEN: n = 353, 31.86%; CIP: n = 492, 44.40%). Only 9 NG isolates were PEN susceptible, while most isolates remained independently susceptible to either AZI (n = 1087, 98.10%) or CTX (n = 1086, 98.01%). Twenty-one isolates, mainly clade 3, were identified as having AZI low-level resistance with MICs ranging between 2 and 32 µg/mL (1.895%). Likewise, 22 isolates, mainly from clade 8, displayed CTX-reduced susceptibility (1.986%).

A minority of isolates displayed no genotypic TET resistance (ie, absence of *tetM* gene and *rpsJ* V57 M; n = 438, 39.53%). Detection of the intermediate TET resistance *rpsJ* V57 M mutation was common (n = 631, 56.95%) and often overlapped with the presence of *tetM* high-level resistance (n = 317, 28.61%), especially for clade 1, 4, and 5 isolates.

### Population Changes as COVID-19 Pandemic–Related Border Restrictions Relaxed

**Figure 3.**
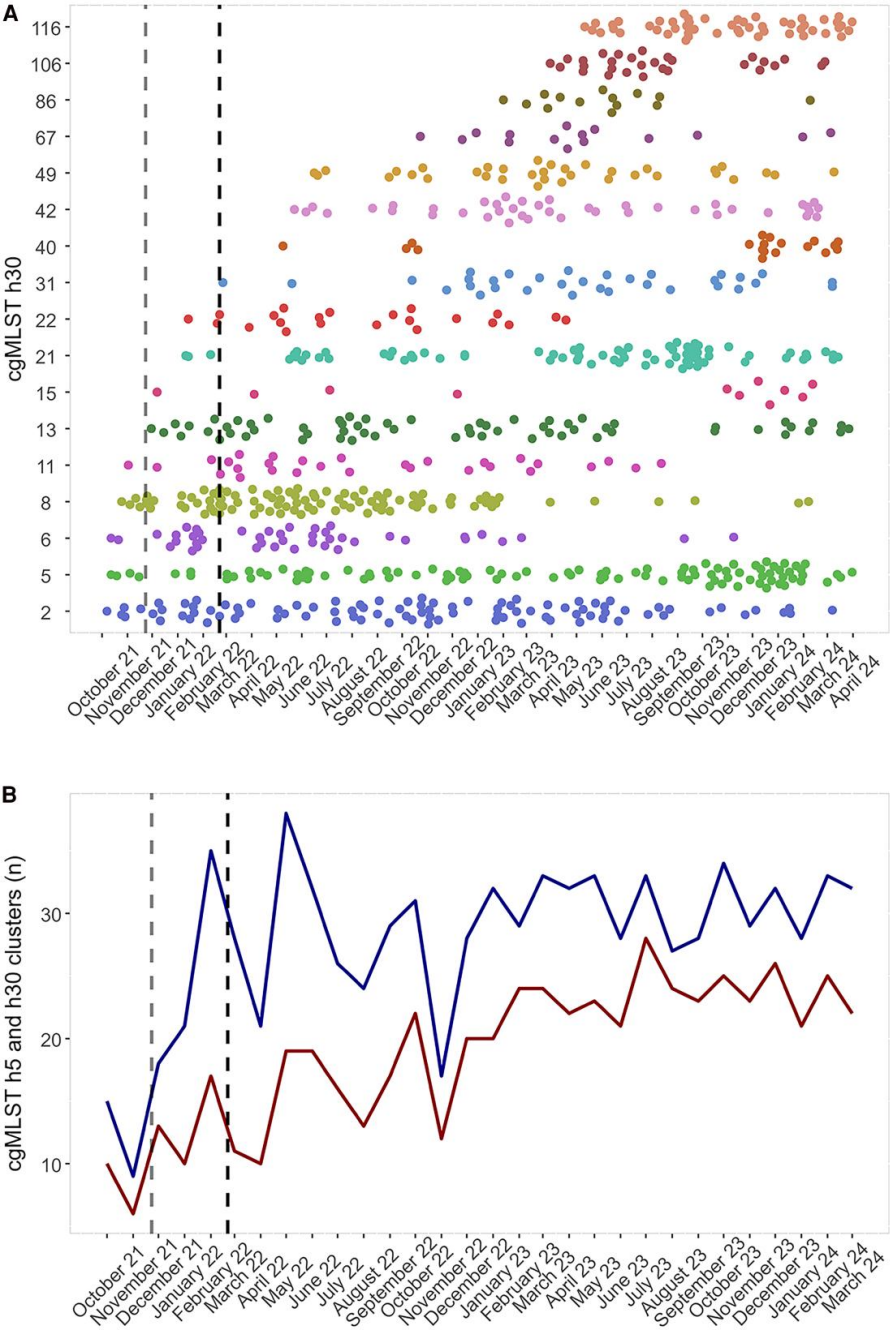
*Neisseria gonorrhoeae* population structure following cessation of COVID-19 interstate and international border restrictions in South Australia. *A*, The top *N gonorrhoeae* strains (n > 10) sequenced during the study period; type was defined by using a cgMLST 30 allele threshold (cgMLST *h*30). *B*, Count of all unique *N gonorrhoeae* cgMLST types defined by using a cgMLST *h*30 (red) or *h*5 threshold (blue). Opening of state borders (23 November 2021) is indicated by the gray dashed line; opening of international borders (21 February 2022) is indicated by the black dashed line. Abbreviation: cgMLST, core genome multilocus sequence type.

NG population changes over time were assessed by hierarchical cgMLST allele thresholds, where 30 or 5 allele differences were used to define broad and highly related clusters (*h*30 or *h*5). Prior to the state border opening, cgMLST diversity was low. Isolates were dominated by 3 cgMLST *h*30 clusters, *cg*C-2, *cg*C-5, and *cg*C-8 (Figure 3*A*), with only 6 *h*30 and 9 *h*5 clusters in total (Figure 3*B*).

The abundance of clusters varied over time, especially after the resumption of interstate travel. Initially, new clusters were detected, including *cg*C-13, *cg*C-15, *cg*C-21, and *cg*C-22, and there was a disproportionate increase in *h*5 vs *h*30 clusters occurring until February 2022 (*h*5 – *h*30: November, Δ3; February, Δ18). This period coincided with expansion of locally dominant *cg*C-2, *cg*C-6, and *cg*C-8 clusters. After February 2022 and the opening of international borders, the proportion of *h*30 and *h*5 clusters fluctuated, and numbers steadily increased until mid- to late 2023 when these began to stabilize. Eight new dominant clusters appeared after the resumption of international travel (*cg*C-31, *cg*C-40, *cg*C-42, *cg*C-49, *cg*C-67, *cg*C-86, *cg*C-106, and *cg*C-116). Locally dominant *cg*C-6 and *cg*C-8 prevalence decreased after January 2023, whereas *cg*C-2 and *cg*C-5 prevalence persisted until December 2023 and March 2024, respectively. Reductions in *cg*C-6 and *cg*C-8 isolates coincided with the expansion of *cg*C-21, *cg*C-31, and *cg*C-67 and the appearance of *cg*C-86, *cg*C-106, and *cg*C-116.

### Molecular Resistance Profiles

**Figure 4.**
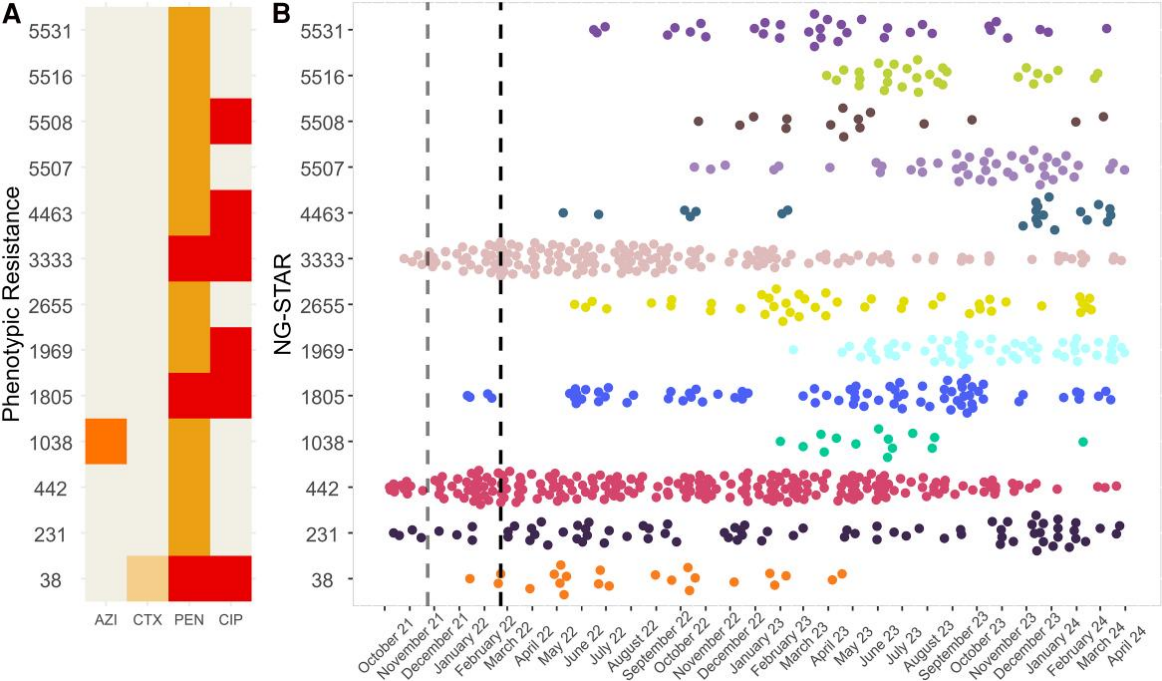
Analysis of the most prevalent NG-STAR sequence types during the study period (n > 10). *A*, The level of phenotypic resistance associated with top NG-STAR sequence types; resistance levels are summarized as follows: sensitive (beige), reduced susceptibility (cream), intermediate (gold), low-level resistant (orange), and resistant (red). *B*, Prevalence of top NG-STAR types detected over time based on sample collection date. Opening of state borders (23 November 2021) is indicated by the gray dashed line; opening of international borders (21 February 2022) is indicated by the black dashed line. Abbreviations: AZI, azithromycin; CIP, ciprofloxacin; CTX, ceftriaxone; NG-STAR, *N gonorrhoeae* Sequence Typing for Antimicrobial Resistance; PEN, penicillin.

Predominant NG-STAR types, their associated molecular AMR markers, and cgMLST *h*30 cluster identity over the study period were assessed (Figure 4, Supplementary Table 1, Supplementary Figure 4). Like cgMLST diversity, the prevalence of *ng*STs fluctuated over time (Figure 4). Prior to the state border opening, most isolates were *ng*ST-231 and *ng*ST-442 (PEN intermediate resistant) or *ng*ST-3333 (PEN and CIP resistant). Immediately after the interstate border opening, an expansion of these existing NG-STAR populations was observed. In mid-January 2021, new dominant NG-STAR types were detected, including *ng*ST-38 (PEN and CIP resistant and CTX reduced susceptibility), *ng*ST-1805 (PEN and CIP resistance), and later *ng*ST-1038 (AZI low-level resistance and PEN intermediate resistance). Interestingly, cgMLST *h*30 clusters were strongly correlated with their *ng*ST (*P* = 2.2e-16, χ^2^ = 113 985). While some *ng*STs were composed of newly introduced monoclonal cgMLST *h*30 clusters (Supplementary Figure 4: *ng*ST-38, *ng*ST-1038, *ng*ST-5508, *ng*ST-5516, and *ng*ST-5531), others were composed of diverse cgMLST populations (*ng*ST-4463, *ng*ST-3333, *ng*ST-2655, *ng*ST-1969, *ng*ST-1805, *ng*ST-442, and *ng*ST-231). There was also evidence of NG-STAR diversification from existing cgMLST *h*30 clusters. Initially, *cg*C-5 was exclusively associated with *ng*ST-231, but after September 2022 it emerged as *ng*ST-5507 due to the replacement of *penA* type II nonmosaic with *penA* type 166 mosaic.

### Epidemiologic Investigations of 2 Priority MDR Clusters After the Border Opening

Further epidemiologic investigations were conducted focusing on 2 newly introduced cgMLST *h*30 monoclonal MDR clusters of interest. These were *cg*C-22 (22 SA cases, 4 VIC cases; ST-7827, *ng*ST-38; CTX reduced susceptibility, PEN resistant/CIP resistant) and *cg*C-86 (14 SA cases, 2 VIC cases; ST-1580, *ng*ST-1038; AZI not susceptible/PEN intermediate resistant).

**Figure 5.**
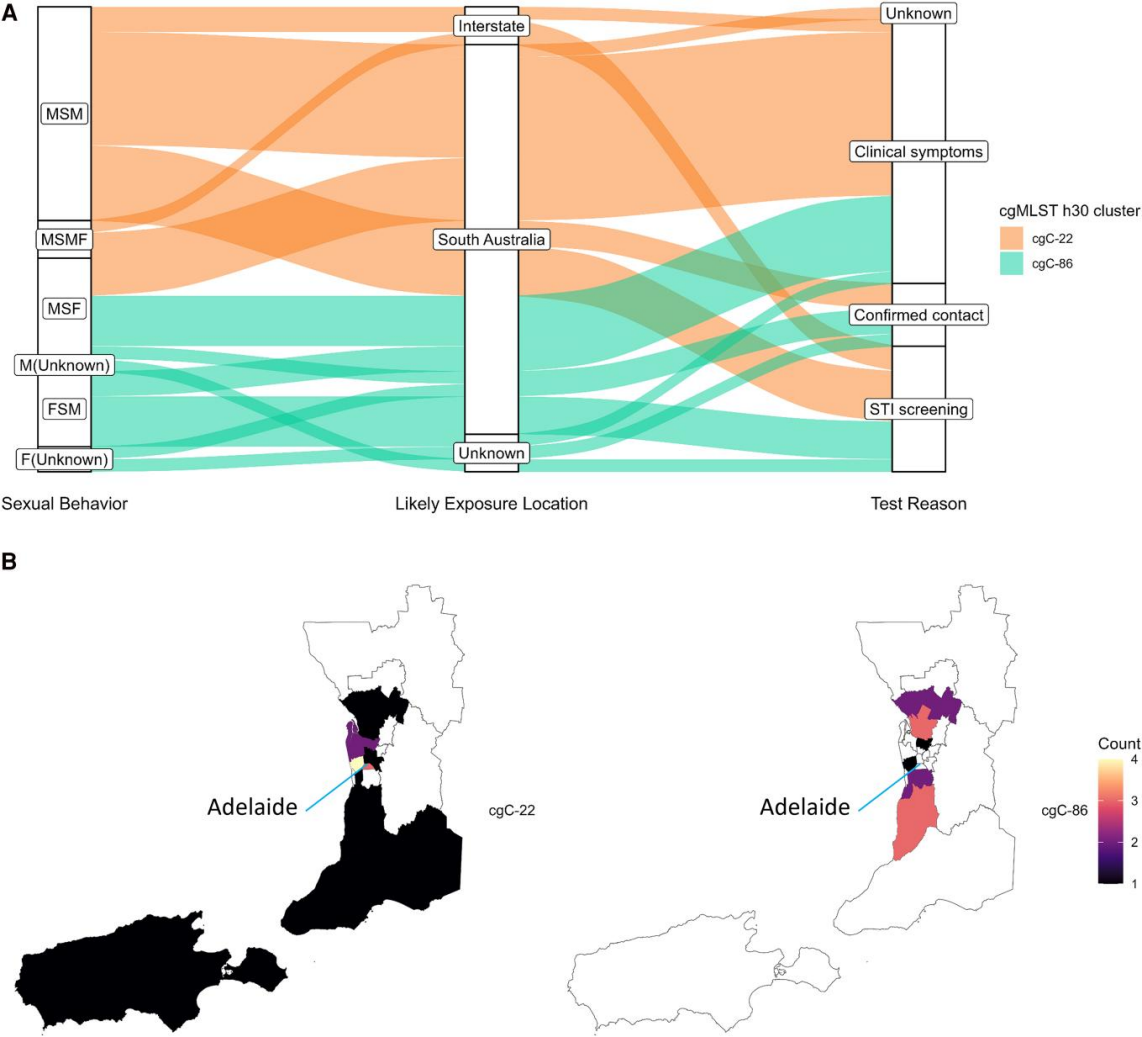
*A*, Epidemiologic characteristics including reported sexual behavior in the previous 12 months, likely exposure location, and testing reason for cases associated with *cg*C-22 and *cg*C-86 monoclonal cgMLST *h*30 clusters. F(Unknown), females with unrecorded sexual behavior; FSM, females who reported having sex only with males; M(Unknown), males with unrecorded sexual behavior; MSF, males who reported having sex only with females; MSM, males who reported having sex only with males; MSMF, males who reported having sex with males and females. *B*, Geographic distribution of *cg*C-22 and *cg*C-86 cases within South Australia determined by residential post code grouped into SA3. Regional-remote and remote areas of the state have been removed from the map to assist with scaling. The location of the capital city (Adelaide) is indicated by the blue line.

SA cases with *cg*C-22 were all male and predominantly men who had sex only with males (MSM; Figure 5*A*). Although most of these cases were reported as locally acquired, there were 3 incidences of interstate acquisition. In contrast, *cg*C-86 SA cases were male (n = 6) and female (n = 8), reported as likely SA acquired, and indicated having sex with partners of the opposite sex or did not disclose sexual behavior. For both clusters, most cases were detected after presenting with clinical symptoms (n = 21), followed by asymptomatic STI screening (n = 10) and close contact screening (n = 4). There were no significant differences in test reason between clusters (symptoms: *P* = .7603, χ^2^ = 0.0931; STI screening: *P* > .99, χ^2^ = 2.113e-31; contact screening: *P* = .5465, χ^2^ = 0.3636).

Differences in the geographic distribution of cases in each cluster were observed (Figure 5*B*). The *cg*C-22 cluster was concentrated in suburbs adjacent to the capital city, with diffuse cases elsewhere in SA. In contrast, the *cg*C-86 cluster was concentrated in northern and southern outer metropolitan areas.

When SA and VIC cgMLST clusters were compared, cases from both SA clusters of interest were related to VIC sequences at the cgMLST *h*30 level, implying shared ancestry. Additionally, a subset of SA cases from *cg*C-22 clustered with VIC sequences at the highly related cgMLST *h*5 allelic difference threshold (Supplementary Table 2 for details). However, none of these SA *cg*C-22 cases clustering with VIC sequences at the *h*5 level reported interstate acquisition, possibly a result of missing epidemiologic links, a low proportion of samples cultured, or incomplete metadata. The 3 *cg*C-22 cases that did report interstate acquisition were either linked with other SA sequences or unlinked at the *h*5 level to all other sequences. All except 3 SA *cg*C-86 cases clustered at the *h*5 level, including 1 isolated case in 2024, likely indicating protracted local transmission. No SA *cg*C-86 cases clustered with VIC sequences at the *h*5 level.

## DISCUSSION

This study revealed the NG genomic epidemiology in SA from the COVID-19 border closure to 2 years following the resumption of interstate and international travel. Overall, sex and age demographics for sequenced cases were consistent with the National Notifiable Disease Surveillance System data for gonococcal infections in SA during these study years [33]. However, sequenced isolates from males were overrepresented as compared with females in the dataset, likely due to factors including the higher rate of asymptomatic infections in females, leading to underdiagnosis, and current guidelines in Australia recommending 3-monthly STI screening for high-risk groups, including MSM [34].

Fortunately, the prevalence of AZI/CTX resistance was low in SA during the study period as compared with recent estimates from Europe and other Australian jurisdictions [4, 35]. These resistances were below the 5% World Health Organization threshold to change first-line treatment and mostly confined to strains that became less prevalent over time. Regardless, ongoing genomic surveillance remains critical to Australia's capacity to monitor and respond to the introduction and expansion of new MDR strain incursions.

The NG population in SA changed markedly following resumption of interstate and international travel. Following a period of restricted travel, there was limited NG genomic diversity coinciding with fewer gonorrhea notifications. After unrestricted interstate travel recommenced, NG genomic diversity rapidly expanded, followed by a phase of steady expansion and then stabilization after the international border opening. NG genomic diversification likely occurred due to a combination of local evolution and new strain introductions following relaxing of travel restrictions. This rebounding of genomic diversity mirrors findings in other countries that NG population diversity was restricted by the implementation of COVID-19 pandemic control measures [5, 6]. Our findings highlight the dynamic nature of the NG genomic population. The Australian jurisdiction Queensland also reported fluctuations in the prevalence of NG strains prior to the COVID-19 pandemic between 2010 and 2015, suggesting that the NG population structure is dynamic irrespective of bottleneck events [36]. The reasons for these fluctuations are likely multifactorial and could include fitness costs associated with certain strains or changes to sexual networks, as well as the effect of public health interventions such as partner notification, contact tracing, follow-up testing, and treatment.

Tetracyclines (eg, doxycycline) are not recommended as treatments for NG; however, since late 2023 the Australasian Society for HIV, Viral Hepatitis and Sexual Health Medicine has recommended doxycycline postexposure prophylaxis (doxy-PEP) for some MSM in Australia [37]. The plasmid-borne *tetM* is a genetic marker for high-level tetracycline resistance in NG [38]. Evidence suggests that while doxy-PEP can reduce the incidence of some STIs, doxycycline can increase tetracycline resistance in NG and other commensal bacteria [39]. Genomic data from this study showed that the plasmid-mediated *tetM* was common in SA at 28.61% and *rpsJ* V57 M mutation was highly prevalent in SA NG at 56.95%. These proportions were similar to 1 New Zealand study of NG isolates from 2018 to 2019 [40]. Despite this, doxy-PEP may facilitate the spread of tetracycline resistance by selection, mobilizable through plasmid conjugation, therefore warranting the continual genomic surveillance of NG tetracycline resistance in the state.

In this study, cgMLST analysis coupled to NG-STAR sequence typing enabled rapid identification of MDR genomic clusters of interest with distinct epidemiologic risk factors, supporting the validity of this approach for routine NG genomic surveillance. These genomic clusters included highly related sequences at the *h*5 allele threshold, indicating recent expansion and possibly identifying local and/or interjurisdictional transmission networks. Future analysis comparing cgMLST cluster thresholds against known contacts, as performed in other studies [28], would be required to validate this method for identification of linked cases. This analysis identified an AZI low-level resistance *ng*ST-1038 *cg*C-86 cluster strongly associated with heterosexual transmission in the northern and southern outer-metropolitan areas. These geographic regions have the highest relative socioeconomic disadvantage aside from rural and remote areas in SA [41]. This socioeconomic disadvantage has the potential to delay outbreak detection due to additional health care access barriers [42, 43], and asymptomatic NG carriage, common in women, could facilitate ongoing undetected transmission [44]. The detection of a single case in 2024 from the *cg*C-86 cluster that was genomically linked at the cgMLST *h*5 level to cases in 2023 supports that there is ongoing undetected transmission of this subclone occurring within SA. The ST of the *cg*C-86 (ST-1580, *ng*ST-1038) was previously detected in Argentina as early as 2016, but the majority of cases were male, indicating that the case demographics can be dynamic [45]. Continued surveillance and public health intervention should be conducted if this strain reemerges.

In contrast, *cg*C-22 cases with reduced susceptibility to CTX were most concentrated near the capital city, with few cases elsewhere in the state. The presence of this cluster indicates that SA is linked to the global spread of the ST-7827, *ng*ST-38 strain, likely originating from East Asia [15]. Sudden emergence of this strain of ST-7827 NG has been reported in Norway and in the Australian jurisdiction of New South Wales [15, 46]. Although international travel was not reported in this cluster, several cases likely acquired their infection interstate or were linked to interstate sequences at the *h*5 level. The majority of cases in this cluster were MSM, consistent with previous findings that this strain is predominantly associated with males [15]. Like other reports of rapid reduction in transmission [15], this strain has been undetected in SA since April 2023. However, we identified several genomically unlinked cases within this group at the cgMLST *h*5 level, suggesting that multiple introductions may have occurred and may reoccur in the future due to widespread global reservoirs.

This study was limited by the number of isolates cultured as compared with total NG notifications. The low culture rates may be due to a preference for molecular detection methods or for collection, culture, and sample transportation practices within SA, therefore affecting culture viability. This low culture success rate could be improved by implementing bedside inoculation practices, and these should be considered in future [47]. Epidemiologic metadata from notifications are subject to transcription and patient reporting accuracy; also, analysis of sex assigned at birth may underrepresent gender-diverse individuals in the dataset. In addition, due to separation of Australia's jurisdictional health agencies, we were unable to analyze contemporaneous sequences from states other than VIC or provide epidemiologic metadata for genomically clustered interstate cases. In the future, a national analysis should ideally be performed to determine Australia-wide strain prevalence and to monitor cross-jurisdictional transmission. Furthermore, this study focused on travel restriction as the predominant COVID-19 prevention measure implemented in SA. This analysis did not assess other restrictions in SA, including lockdowns (10 days total), venue closures, limited size of social gatherings, or behavioral changes during this time due to their inconsistent implementation [48].

Collectively, this study demonstrates fluctuations in the NG population and associated AMR determinants during and following Australia's COVID-19–related travel restrictions. This was likely driven by bottleneck effects, local strain diversification, and the introduction of newly detected strains linked to international and interstate travel. It also highlights the importance of genomic surveillance to effectively monitor the evolution of AMR in gonorrhea by enabling elucidation of the genetic mechanisms driving the spread of resistance. Moreover, it finds that cgMLST coupled with NG-STAR can be used to support targeted public health interventions for MDR gonorrhea.

## Supplementary Material

jiag097_Supplementary_Data

## Supplementary Data

Supplementary materials are available at *The Journal of Infectious Diseases* online (http://jid.oxfordjournals.org/). Supplementary materials consist of data provided by the author that are published to benefit the reader. The posted materials are not copyedited. The contents of all supplementary data are the sole responsibility of the authors. Questions or messages regarding errors should be addressed to the author.
