# Supplementary material for: Increased Diversity and Introduction of Multidrug-Resistant Strains of Neisseria gonorrhoeae Following Cessation of COVID-19 Pandemic–Related Travel Restrictions: An Observational Genomic Epidemiologic Study
Source: J Infect Dis. 2026 Feb 12;233(5):e1130–40. doi: 10.1093/infdis/jiag097 (PMC13175631; doi:10.1093/infdis/jiag097)
Supplement: jiag097_Supplementary_Data [file jiag097_supplementary_data.zip › Supplementary_Figure1_cgMLST_loci.pdf]

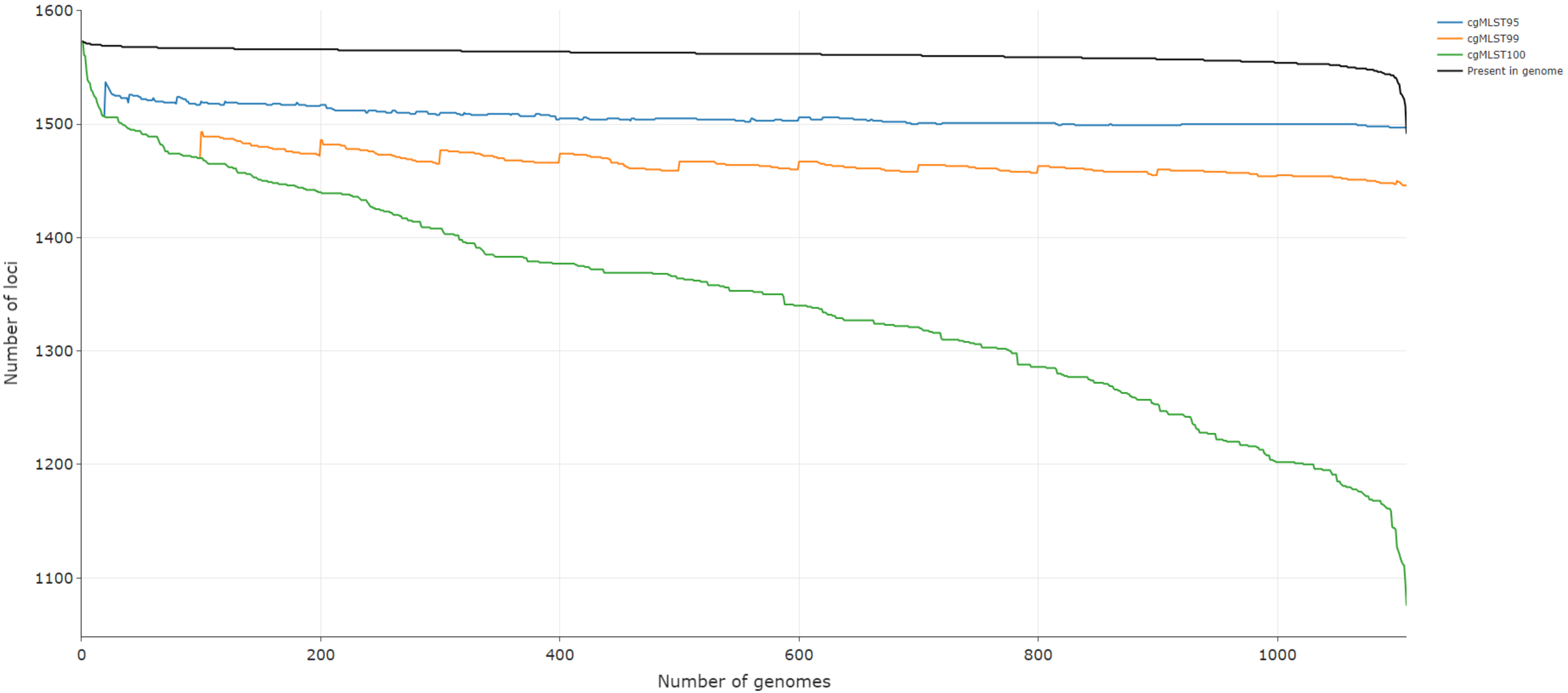

Supplementary Figure 1: The number of common loci present in 1,108 SA sequences that passed quality assessment at 95%, 99% and 100% allele thresholds.
