# Supplementary material for: Increased Diversity and Introduction of Multidrug-Resistant Strains of Neisseria gonorrhoeae Following Cessation of COVID-19 Pandemic–Related Travel Restrictions: An Observational Genomic Epidemiologic Study
Source: J Infect Dis. 2026 Feb 12;233(5):e1130–40. doi: 10.1093/infdis/jiag097 (PMC13175631; doi:10.1093/infdis/jiag097)
Supplement: jiag097_Supplementary_Data [file jiag097_supplementary_data.zip › Supplementary_Figure4_topNG-STAR_cgMLST_h30.pdf]

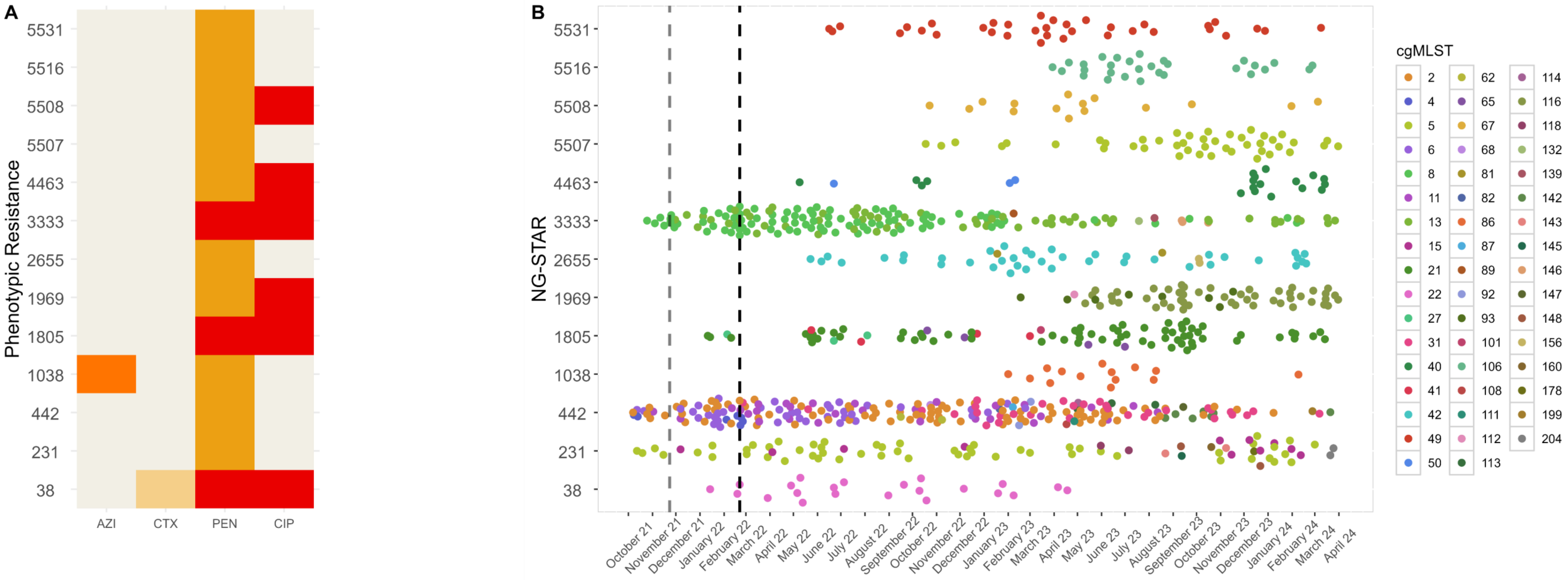

Supplementary Figure 4: cgMLST h30 cluster diversity amongst NG-STAR types with >10 isolates sequenced during the study period. A) The level of phenotypic resistance associated with top NG-STAR types, resistance levels are summarised as "Sensitive" (beige), "Reduced Susceptibility" (pale yellow), "Intermediate" (gold), "Low-Level Resistant" (orange), "Resistant" (red). B) Prevalence of top NG-STAR types detected over time based on sample collection date and coloured by cgMLST h30 cluster identity. Cessation of COVID-19 restrictions in SA is indicated by the grey dashed line.
